# Purine Acquisition and Synthesis by Human Fungal Pathogens

**DOI:** 10.3390/microorganisms5020033

**Published:** 2017-06-08

**Authors:** Jessica L. Chitty, James A. Fraser

**Affiliations:** 1 Australian Infectious Diseases Research Centre, School of Chemistry & Molecular Biosciences, the University of Queensland, St Lucia, Queensland 4072, Australia; j.chitty@uq.edu.au; 2 Institute for Molecular Bioscience, the University of Queensland, St Lucia, Queensland 4072, Australia

**Keywords:** fungal pathogens, purines, nitrogen, degradation, salvage, synthesis

## Abstract

While members of the Kingdom Fungi are found across many of the world’s most hostile environments, only a limited number of species can thrive within the human host. The causative agents of the most common invasive fungal infections are *Candida albicans*, *Aspergillus fumigatus*, and *Cryptococcus neoformans*. During the infection process, these fungi must not only combat the host immune system while adapting to dramatic changes in temperature and pH, but also acquire sufficient nutrients to enable growth and dissemination in the host. One class of nutrients required by fungi, which is found in varying concentrations in their environmental niches and the human host, is the purines. These nitrogen-containing heterocycles are one of the most abundant organic molecules in nature and are required for roles as diverse as signal transduction, energy metabolism and DNA synthesis. The most common life-threatening fungal pathogens can degrade, salvage and synthesize de novo purines through a number of enzymatic steps that are conserved. While these enable them to adapt to the changing purine availability in the environment, only de novo purine biosynthesis is essential during infection and therefore an attractive antimycotic target.

## 1. The Diversity of Fungi and the Environments They Inhabit

Federico Cesi, founder of the *Accademia dei Lincei* and a keen observer of his local environment, first attempted the scientific classification of organisms. “Imperfect” plants, particularly fungi, fascinated Cesi; his colleague Galileo Galilei constructed a microscope to help him observe these organisms in great detail, and Cesi commissioned hundreds of drawings of mushroom species collected from Rome and southern Umbria until his death in 1630 [1]. The Italian priest and botanist Pier Antonio Micheli later continued Cesi’s classification. His most notable work *Nova Plantarum Genera* documented 1400 new “plant” species collected from around Europe, of which 900 were fungi or lichens and included the first documented human fungal pathogen [2]. A few decades later, Swedish botanist and zoologist Carl Linné made a significant contribution to modern taxonomy by classifying organisms from around the globe in his seminal work *Systema Naturae*, although fungal species were poorly addressed in this publication [3]. Almost 100 years later, mycologists Christian Hendrik Persoon and Elias Magnus Fries addressed these shortcomings by classifying fungi sent by leading scientists from around the world [4,5,6]. Combined, these extensive works that took place over four centuries identified thousands of fungi that expanded well beyond Cesi’s Italian mushrooms to include more diverse species inhabiting a wide range of environments. However, it was not until 1969 that the Fungi were classified as their own kingdom and not a subset of plants [7,8]. With the aid of genomic sequencing, the number of species identified now numbers over one million and these are believed to be just the tip of the iceberg, accounting for an estimated 5–7% of species with many environments largely unsampled [9,10]. 

Species from the kingdom Fungi differ greatly in habitat, morphology and nutrient requirements. As heterotrophs, these organisms digest organic molecules such as proteins, polysaccharides and nucleotides, and are often found in nutrient rich environments. In contrast, many fungi can survive in extreme conditions. *Aspergillus sydowii* inhabits deep-sea hydrothermal vents more than 700 meters below sea level where temperatures reach almost boiling point [11]. *Penicillium chrysogenum* can be found in the Atacama Desert, where it has been hyper arid for at least three million years [12]. *Nadsoniella nigra* var. *hesuelica* survives periodic freezing and thawing in Antarctica [13]. Other species survive in a very different extreme environment—the human host —where fungal pathogens must be heat tolerant, resilient to immune defenses and scavenge nutrients that can be difficult to acquire [14].

Of the fungal pathogens affecting humans, three pose the most consistent major threat worldwide: *Aspergillus fumigatus*, *Candida albicans* and *Cryptococcus neoformans*, causing aspergillosis, candidiasis and cryptococcosis, respectively. Even with the best available antifungal treatment in developed countries, low efficacy, toxicity and resistance is a major contributor to the high mortality associated with these invasive fungal infections [15,16]. Each preferentially infects specific sites in patients, from the lung in pulmonary aspergillosis to the brain in cryptococcal meningoencephalitis and the blood stream in systemic candidiasis, yet all employ similar mechanisms to acquire sufficient nutrients to survive and establish an infection [17,18,19]. 

Unlike *C. neoformans* and *A. fumigatus*, which are often found in soil, guano and decaying matter [20,21,22,23], *C. albicans* is a commensal species, commonly found in the gastrointestinal tract and on mucocutaneous surfaces [24,25]. While these environments are vastly different, one important class of biological compounds consistently present are the purines.

To expel excess nitrogen, bird excreta contains high concentrations of the insoluble hetrocyclic compound uric acid, a strategy that reduces water loss compared to the mammalian excretion of nitrogen as its soluble derivative urea [26]. Fresh plant matter such as cauliflower has a purine content of approximately 0.4 mg/g [27]; when living organisms, such as plants, decompose, purines become enriched in soil and so are available to fungi such as *C. neoformans* and *A. fumigatus* [28]. Furthermore, the different tissues of the live human host also vary significantly in the context of purines. The concentrations of purines in the gastrointestinal tract inhabited by *C. albicans* are dependent on the host’s diet, whereas the cerebral spinal fluid to which *C. neoformans* disseminates is a particularly purine-poor environment [29,30,31].

## 2. Purines and Their Role in the Cell

Purine, a term coined by Emil Fisher in 1884 after he synthesized a novel compound from “pure urine”, is a molecule composed of one six and one five-membered nitrogen-containing ring fused together. The addition of at least one phosphate to this molecule makes it a nucleotide, a biochemically important component of the cell [32]. These molecules are essential to DNA and RNA biosynthesis, energy metabolism and signal transduction, and are the most widely occurring nitrogen-containing heterocycle in nature [33]. Purines are also predicted to have been amongst the first organic compounds synthesized by abiotic chemistry on the early earth; adenine, the nucleobase of adenosine triphosphate (ATP), is proposed to have been formed during the prebiotic era by the condensation of five hydrogen cyanide molecules [34,35].

## 3. Purines as a Nitrogen Source

Defects in the degradation of purines for the excretion of excess nitrogen were first implicated to play a role in disease in 1848 by Alfred Garrod. However, the biochemical process in a microbe of degrading purines to acquire nitrogen required for growth was not studied until 1853 when Friedrich Wohler investigated this process in an unidentified yeast species. Nitrogen is a major component of a number of molecules, including amino acids, pyrimidines, and purines, and is essential for life. Fungi are known for their ability to use a wide range of nitrogen sources via a range of catabolic enzymes [36,37].

**Figure 1:**
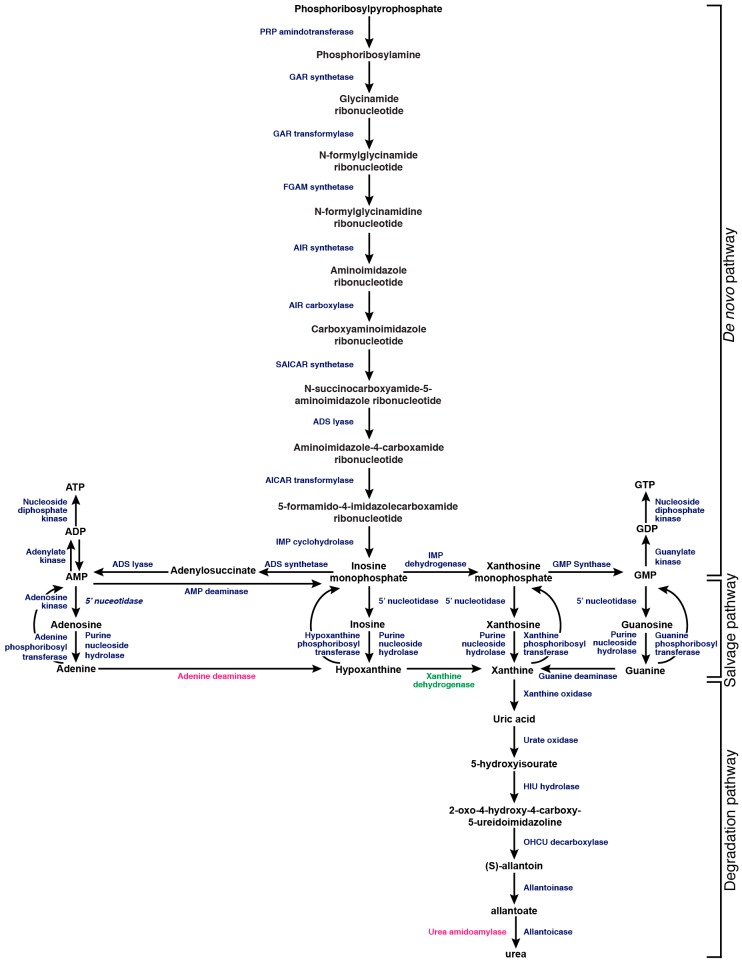
Blue represents enzymes found in *C. albicans*, *A. fumigatus*, and *C. neoformans*. Pink represents an enzyme found in both *C. albicans* and *A. fumigatus*. Green represents an enzyme found in *A. fumigatus* only. Abbreviated enzyme names: PRP (Phosphoribosylpyrophosphate) amidotransferase, GAR (glycinamide ribotide) synthetase, GAR (phosphoribosyl-glycinamide) transformylase, FGAM (formylglycinamidine-ribonucleotide) synthetase, AIR aminoimidazole ribotide) synthetase, AIR (Phosphoribosylaminoimidazole) carboxylase, SAICAR (N-succinyl-5-aminoimidazole-4-carboxamide ribotide) synthetase, ADS (adenylosuccinate) lyase, AICAR (5-aminoimidazole-4-carboxamide ribonucleotide) transformylase, IMP (inosine monophosphate) cyclohydrolase, ADS (adenylosuccinate) synthetase, IMP (inosine monophosphate) dehydrogenase, GMP (guanine monophosphate) synthase.

The process of utilizing purines as a nitrogen source proceeds via the degradation of xanthine to uric acid by xanthine oxidase. Uric acid can then be sequentially degraded by a further five enzymatic activities to produce ammonia (Figure 1). Depending on the fungal species, the entry point for this degradation pathway varies. For example, during its evolution into a facultative anaerobe *Saccharomyces cerevisiae* lost oxygen-dependent urate oxidase, but it can still use allantoin or allantoate as a nitrogen source. In contrast, the fungal pathogen *C. neoformans* can utilize uric acid as a sole nitrogen source [38,39,40]. 

The importance of maintaining a fully functional degradation pathway is not true for all fungi; some plant colonizing species of fungi such as *Piriformospora indica* lack this ability [41]. The Pneumocystis pneumonia-causing fungus *Pneumocystis jirovecii* also lacks the catabolic enzymes required for the degradation of purines, although this is the only human fungal pathogen known to do so [42]. 

## 4. Salvaging Purines

As well as breaking down the purines obtained from the environment to serve as a nitrogen source, fungi also scavenge these essential nutrients for metabolic processes. Small molecules such as nucleotides are detected by plasma membrane-localized sensors to be transported across the plasma membrane to be used in nucleotide biosynthesis [43]. A number of proteins have been identified in fungi that transport purines. Three distinct nucleobase-specific transporter classes exist: nucleobase-ascorbate transporter (NAT) families 1 and 2, the nucleobase cation symporter family 1 (NCS1) and the AzgA-like family. These are all secondary active transporters as they catalyze the transport of two chemical species, a purine and a proton, in the same direction [44], Once scavenged nucleotides are transported into the cell via these dedicated transporters, they are available for incorporation into the salvage pathway. The principal enzymes responsible for the interconversion of purines are hypoxanthine xanthine guanine phosphoribosyltransferase (HXGPT) and adenine phosphoribosyltransferase, enzymes that transfer a 5-phosphoribosyl group to a purine to create the corresponding nucleotide.

## 5. Synthesizing Purines

**Figure 2:**
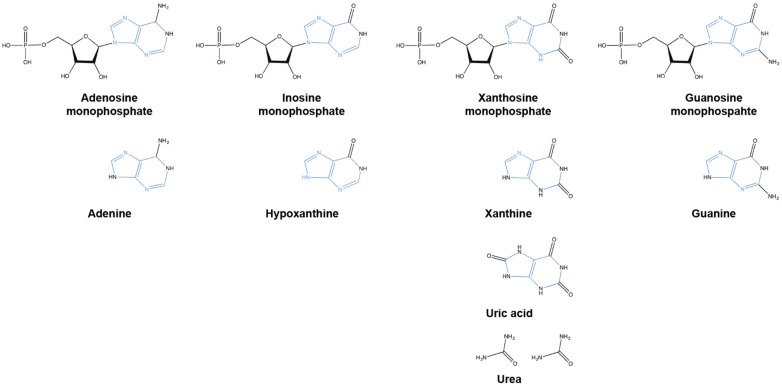
Structures of the key intermediates involved in de novo and salvage pathways containing a purine ring (blue) and the resultant non-purine breakdown product urea.

Fungi may not always be able to salvage sufficient purines from the environment; in many cases nucleotides must be synthesized de novo from precursor molecules. Acquiring purines from the environment is energetically favorable compared to de novo biosynthesis, which requires 14 enzymatic activities and a number of cofactors for the magnesium-dependent generation of either ATP or guanosine triphosphate (GTP) from phosphoribosyl pyrophosphate. Inosine monophosphate (IMP) biosynthesis requires the acquisition of ammonia from two molecules of l-glutamine, ligation of l-aspartate, and hydrolysis of four ATP molecules, with two 10-formyl-THF formyl donors. For the synthesis of ATP from IMP (Figure 2), an additional l-aspartate molecule is ligated, and one GTP and two ATP molecules are hydrolyzed. The synthesis of GTP from IMP (Figure 2) requires an additional molecule of one l-glutamine, hydrolysis of three ATPs and the hydride transfer from one nicotinamide adenine dinucleotide (NAD^+^) molecule. In total, in order for de novo synthesis to occur, 10 molecules of ATP are hydrolyzed per molecule of AMP synthesized, and 11 for GMP [45,46]. It is estimated that 10^7^ ATP molecules are used per second per *S. cerevisiae* cell; therefore, the steady supply of purine nucleotides is essential for survival. While scavenging these purines is the most energy efficient strategy, having an intact de novo purine synthesis pathway is highly advantageous to fungi that inhabit environments with varying concentrations of purines [46].

Some species of fungi have lost the ability to synthesize purines and rely solely on the salvage of nucleotides from their environment. The Microsporidia have lost a number of enzymatic activities required for the de novo synthesis of purines, meaning the complete process is no longer possible in these obligate parasites [47,48]. However, the high expression of nucleoside diphosphate kinase required for the phosphorylation of adenosine and guanosine suggests that aspects of purine biosynthesis still play an important role in the metabolism of these parasites [47].

## 6. Purine Metabolism in *Candida Albicans*

While records detailing the symptoms of oral candidiasis date back to 400 B.C., for many centuries, these were thought to originate from the host rather than an infectious agent [49,50]. In 1771, Rosen von Rosenstein identified an invasive fungal pathogen as the causative agent of this disease, and, in 1847, the French mycologist Charles Philippe Robin classified it as *Oidium albicans* [51,52]. Almost a century later, Christine Marie Berkhout reclassified it under the current genus *Candida* [52].

The commensal pathogen *C. albicans* is a frequent member of the gut microbiota; in healthy individuals, it is observed in approximately 40% of the population [25]. For those that do not have an intact immune system, this pathogen poses a major threat and is the leading cause of hospital-acquired bloodstream infections, with those in intensive care units being most at risk [53]. The switch from unicellular commensal yeast to pleiomorphic invasive pathogen is driven by multiple environmental cues. In vitro, this can be induced by changes in pH, temperature, CO_2_ concentration, serum and many other factors [54,55,56,57].

The yeast form of *C. albicans* commonly found in the gastrointestinal tract has plentiful access to nutrients, including proteins, carbohydrates, fats and nucleotides such as purines. The concentration of available purines varies depending on the diet of the host (Table 1) with foods high in purines such as seaweed containing millimolar concentrations, and foods low in purines such as carrots containing nanomolar concentrations [58]. Prior to purine absorption by the host in the small intestine—in particular, in the mucosa of the duodenum—these purines are available to be scavenged by the gut microbiota [59]. In its pathogenic form, *C. albicans* is found in the bloodstream where it causes candidemia; the available purines in the blood are in the micromolar concentration range (Table 1) [60]. 

Enzymes required for the degradation of purines into ammonia have not been well characterized in *C. albicans*. BLASTp analyses using *C. neoformans* orthologs revealed that genes predicted to encode the majority of purine degradation components are present in *C. albicans*. Six enzymes required for the sequential breakdown of xanthine to ammonium were identified: xanthine oxidase (C2_00180C) for the conversion of xanthine to uric acid, 5-hydroxyisourate (HIU) hydrolase (C2_08460C) for the conversion HIU to 2-oxo-4-hydroxy-4-carboxy-5-ureidoimidazoline (OHCU), OHCU decarboxylase (C3_01620W) for the conversion of OHCU to (S)-allantoin, allantoinase (C3_00180C) for the conversion of allantoin to allantoate, and allantoicase (C2_00630C) converting allantoate to urea. For the final step of hydrolyzing urea to ammonia, *C. albicans* encodes a urea amidolyase (C1_04660W) that carries out two steps: carboxylation of urea to urea-1-carboxylate, followed by hydrolysis to two molecules of ammonia. In addition, a zinc cluster transcription factor exclusively found in fungi has been identified as playing a role in purine catabolism in *C. albicans*. Ppr1 regulates uracil degradation in *S. cerevisiae* but plays a different role in *C. albicans*, where it is involved in the regulation of allantoin degradation [64]. 

Salvage of nitrogenous compounds by *C. albicans* from the environment is essential in the production of virulence factors such as adherence to the host tissue, hyphal morphogenesis, and release of ammonia to counteract acidification of the phagolysosome [65]. UV microscopy has shown that, in vitro, *C. albicans* actively takes up the purines guanine and adenine within a few hours from the growth medium. The concentrations of purines within the vacuole of the cell became supersaturated, suggesting that when purines are available, the fungus scavenges all it can [66]. The consumption of extracellular nucleotides in *C. albicans* occurs via their hydrolysis to nucleosides by ecto-enzymes attached to the cell membrane that actively import purines into the cell [67]. 

While the enzymes of the purine salvage pathway have not been characterized in this species, BLASTp analyses using *C. neoformans* orthologs revealed the presence of a gene predicted to encode adenine phosphoribosyl transferase (C2_01430W), suggesting that *C. albicans* can covert adenine to AMP. A likely member of the phosphoribosyl transferase family (C2_02740C) responsible for the conversion of one or more of hypoxanthine, xanthine and/or guanine into their respective phosphorylated nucleotides is also present. Other enzymes involved in the interconversion of purines are anticipated in *C. albicans*; a BLASTp analysis using *S. cerevisiae* orthologs identified genes predicted to encoded adenine deaminase (C2_03360W) and guanine deaminase (C7_00670W) for the conversion of adenine to hypoxanthine and guanine to xanthine. 

While only a few of the genes required for de novo purine biosynthesis have been characterized in *C. albicans*, BLASTp analysis using *C. neoformans* orthologs reveals genes thought to encode the ten enzymatic activities required for the conversion of phosphoribosyl pyrophosphate to IMP: PRP transferase (C1_07710C), GAR synthetase/AIR synthetase (C1_07890C), GAR transformlyase (C2_03090C), FGAM synthetase (CR_04740C), AIR carboxylase (C3_04520C), SAICAR synthetase (CR_06150C), ADS lyase (CR_06150C) and AICAR transformylase/inosine cyclohydrolase (ATIC) (CR_04090C). Additionally, the four enzymatic activities for synthesis of ATP from IMP (ADS synthetase (C1_09640W), ADS lyase (CR_06150C), adenylate kinase (C6_01910W) and nucleotide diphosphate kinase (C5_02890W)) and the four activities for GTP synthesis from IMP (IMP dehydrogenase (C2_06390C), GMP synthase (C1_09490C), guanylate kinase (C503790W), and nucleoside diphosphate kinase (C5_02890W)) are also predicted to be encoded by genes identified in *C. albicans*.

Mutation and deletion studies of some of the genes encoding the enzymatic activities required for de novo purine biosynthesis have been carried out in *C. albicans*. Mutants found to produce red pigmentation were hypothesized to encode AIR carboxylase and SAICAR synthetase based on *S. cerevisiae* studies identifying the pigmentation as a result of the accumulation of oxidized and polymerized AIR in the vacuole [68]. Subsequent targeted gene disruption of the *ade2* gene encoding AIR carboxylase in *C. albicans* results in reduced virulence in a murine candidiasis model [69]. The strain was unable to proliferate in human serum unless supplemented with exogenous adenine, and, although not completely avirulent, exhibited a 100-fold attenuation of virulence [69]. 

The deletion of genes encoding two other enzymes of the de novo purine biosynthesis pathway in *C. albicans* has also been performed. The genes *ADE8* and *GUA1* encoding the enzymes GAR transformylase and GMP synthase have been deleted and in vivo growth assays for both strains showed they were unable to grow on media without supplementation of exogenous purines; adenine was required for the *ade8* mutant and guanine was required for the *gua1* mutant [70,71,72]. Both of these deletion strains were hypersensitive to the purine biosynthesis inhibitors methotrexate and 6-azauracil [71]. In a candidiasis model of infection, the *gua1* strain is avirulent [70].

## 7. Purine Metabolism in *Aspergillus Fumigatus*

Historically, the *Aspergillus* molds have been recognized as a genus since 1729 [2]. They have been attributed to infection since the French revolution, and the species *fumigatus* known to frequently cause aspergillosis since the early 1900s [73,74]. More recently *A. fumigatus* has become recognized as the most prevalent airborne fungal pathogen, commonly causing severe or fatal infections in immunocompromised individuals [75,76,77].

The asexual conidia of *A. fumigatus* are produced in abundance and inhaled by animals and humans on a regular basis. In healthy individuals, these are cleared by the innate immune system; however, in an immunodeficient individual, they pose a significant risk. Invasive aspergillosis is frequently observed in cancer and transplant patients, accounting for 10–25% of life-threatening opportunistic infections in leukemia treatment centers and 15–25% in transplant units [77,78,79,80,81,82,83].

Like many decomposers, *A. fumigatus* is commonly found in soil where organic matter provides plentiful nutrients. Purine availability varies considerably in this niche depending on a number of factors. Investigation of purine composition of soil has identified that humic acids (the principle component of soil humus) are richer in the purines guanine and adenine than the pyrimidines cytosine, thymine and uracil. The concentration of purines in dry soils have been shown to range from 21 to 138 μg per gram and the concentration distribution is consistent within soils: guanine is the most abundant, followed by cytosine, adenine, thymine and the least abundant, uracil [63]. Purine concentrations also vary greatly in plant life that will eventually become part of the diet of saprobes; the legume *Alysicarpus vaginalis* and the Jujube fruiting tree species *Ziziphus jujube* and *Ziziphus mauritiana* have been determined to have average purine concentrations of 0.005 to 2.6 μg/mL of guanine and 0.002 to 1.2 μg/mL of adenine (Table 1) [61,62]. 

Compared to this, the lung of an infected individual is a vastly different environment. The small size of the conidia of *A. fumigatus* (2–3 μm) allows them to enter the respiratory tract, descend to the aveoli and bind to surfactant proteins to be endocytosed by epithelial cells [78,84,85]. In the lungs, host-produced extracellular ATP plays a role as an endogenous signaling molecule involved in inflammation [86]. Once in the bloodstream, *A. fumigatus* encounters guanine at a concentration of 97 μM and adenine at 0.2 μM (Table 1).

While purine metabolism has not been well characterized in *A. fumigatus*, more extensive characterization of the pathway in *Aspergillus* species has been carried out in *Aspergillus nidulans*, with all enzyme-encoding genes believed to be associated with degradation, salvage, and de novo biosynthesis of purines identified in this species [87,88,89,90]. BLASTp analysis using *A. nidulans* orthologs revealed that the majority of purine degradation components are also likely present in *A. fumigatus. A. nidulans* encodes a second enzyme, xanthine α ketoglutarate dependent dioxygenase, for the conversion of xanthine to uric acid; this was not identified in *A. fumigatus* by BLASTp analysis but the alternative enzyme xanthine oxidase (Afu2g10520) (which is also present in *A. nidulans*) was identified.

BLASTp analyses using *A. nidulans* orthologs revealed the genes predicted to encode enzymes of the salvage pathway in *A. fumigatus*, including hypoxanthine xanthine guanine phosphoribosyl transferase (Afu4g04550) and adenine phosphoribosyl transferase (Afu7g02310), suggesting that *A. fumigatus* can convert hypoxanthine to IMP, xanthine to XMP, guanine to GMP, and adenine to AMP, respectively. In addition to phosphoribosyl transferase enzymes, *A. fumigatus* is predicted to encode adenine deaminase (Afu8g02860) and xanthine dehydrogenase (Afu4g11220) for the conversion of adenine to hypoxanthine and hypoxanthine to xanthine. The adenine deaminase enzyme encoded by the *nadA* gene is involved in the conversion of AMP to IMP and can be considered as a degradation or a salvage enzyme. In *A. nidulans*, adenine deaminase is essential for the utilisation of adenine as a sole nitrogen source, and, unlike other enzymes required for purine degradation, its expression is not supressed by ammonium, perhaps reflecting an increase in purine interconversion when grown in favourable conditions [91].

BLASTp analyses of the characterized genes of de novo purine biosynthesis from *A. nidulans* predicted genes encoding the ten enzymatic activities required for the conversion of phosphoribosyl pyrophosphate to IMP to be present in *A. fumigatus*, as are the four enzymatic activities for synthesis of ATP from IMP and the four activities for synthesis of GTP from IMP. The deletion of the purine biosynthesis GMP synthase-encoding *guaA* gene in *A. fumigatus* has shown that the strain is unable to grow on media lacking exogenous guanine, and, in a murine model of infection, the *guaA* deletion mutant was avirulent [70]. Computational modeling has supported the hypothesis that these purine biosynthesis enzymes could serve as potential drug targets in *A. fumigatus* and the related species *Aspergillus niger* [92].

## 8. Purine Metabolism in *Cryptococcus Neoformans*

The basidiomycete yeast *Cryptococcus neoformans* was first identified in 1894 by Sanfelice in peach juice and associated with disease shortly after, identified from lesions from the tibia in a 31-year-old patient [93,94]. The division of higher fungi or Basidiomycota is important for the effective breakdown of organic compounds in the environment. Their coevolution with woody plants for over 350 million years has given rise to many species possessing ligno-cellulytic enzymes that digest plant cell walls. This digestive process is essential in the formation of soil humus [95,96,97]. The soil humus contains varying levels of purines, highly dependent on the flora of the area (Table 1).

As well as soil, *C. neoformans* is commonly associated with bird guano. Unlike mammals, some species require excess nitrogen to be converted to uric acid for its excretion rather than urea; the uric acid cycle requires more energy but conserves water, which, for many organisms such as birds, is more important [98,99]. Bird excreta, or guano, has long been valued for its fertilizing properties. Ancient South American civilizations added this fertilizer to enrich soil and improve crops, risking their lives to sail 21 km off the coast of Pisco to the guano-rich Chincha Islands [100]. This ancient tradition reached Europe through the exploration of Alexander von Humboldt, leading to a time that became known as the guano boom [101]. Chemists such as Gustav Magnus analyzed the nitrogen content to determine the prices that guano should be sold. He reported an acid precipitation on the guano material and found a novel compound now known as guanine to be in high concentrations [102]. Bird guano is also high in uric acid, the ingredient responsible for the bird guano-associated damage of buildings, particularly limestone. Since the identification of guanine from guano, no quantitative analysis has been done to identify the specific concentration of purines in this substrate. 

*C. neoformans* has been associated with bird guano since the 1960s and has since been identified worldwide from bird droppings in a range of locations [21,22]. Pigeon guano medium supports the growth of *C. neoformans* and the production of a key virulence factor, melanin. In addition, *C. neoformans* is able to undergo its sexual cycle on pigeon guano, supporting the theory that pigeon guano is the ecological niche of this fungal pathogen, as it can complete its life cycle solely in this environment [103].

Unlike *Aspergillus* species that can use a wide range of nitrogen sources, *C. neoformans* is more limited and restricted to ammonium, amino acids and purines [37]. All genes encoding the predicted enzymes of the purine degradation pathway in *C. neoformans* have been characterized. Six enzymatic reactions are required for the breakdown of xanthine to ammonium and are as follows: urate oxidase for oxidation of urate to HIU (CNAG_04307, *URO1*), HIU hydrolase (CNAG_06694, *URO2*) hydrolyzing HIU to OHCU, OHCU decarboxylase (CNAG_00639, *URO3*) converting OHCU to (S) allantoin, allantoinase (CNAG_00934, *DAL1*) hydrolyzing allantoin to allantoate, allantoicase (CNAG_01108, *DAL2*) converting allantoate to urea, and finally urease (CNAG_05540, *URE1*) hydrolyzing urea to ammonium [104]. Each of the genes identified to encode these enzymes has been sequentially deleted and characterized [104]. None of these deleted genes affected production of the virulence factors capsule or melanin, nor initiation of the *C. neoformans* sexual cycle [104]. In a murine inhalation model of cryptococcosis, only urease, the final enzyme of the pathway for the hydrolysis of urea to ammonia, is required for pathogenesis [105,106,107]. 

The salvage pathway in *C. neoformans* consists of a number of enzymes that can interconvert purine intermediates. The nucleoside hydrolases are required for the hydrolysis of nucleotides to nucleosides; hypoxanthine-xanthine-guanine phosphoribosyltransferase (CNAG_02546, *HPT1*) (HXGPRT) and adenine phosphoribosyltransferase (CNAG_01390, *APH1*) phosphorylate the nucleotides hypoxanthine, xanthine, guanine, and adenine to IMP, XMP, GMP and ATP, respectively [108,109]. The phosphoribosyltransferases have been studied in this organism, and the deletion of the genes encoding these enzymes did not result in any phenotypic differences from the wild-type, nor affect virulence in a murine model of cryptococcosis suggesting purine salvage is not important during the infection process [108,109].

Like *C. albicans* and *A. fumigatus*, *C. neoformans* encodes ten enzymatic activities for the conversion of phosphoribosylpyrophosphate to IMP. Additionally, four enzymatic activities are required for IMP to be converted to GMP and four enzymatic activities for its conversion to AMP. Deletion of the gene encoding AIR carboxylase (CNAG_02294, *ADE2*) showed the identical phenotype to *C. albicans* and *S. cerevisiae* of red pigmentation produced and similarly led to the pathway’s investigation as a potential antifungal drug target. The *ade2Δ* mutant in a murine inhalation model and rabbit cryptococcal meningitis model was avirulent [110,111]. Enzyme kinetic assays using recombinantly purified protein revealed differences in activity between *C. neoformans* and *Gallus gallus* AIR carboxylase, suggesting that this could be a novel target of inhibition against the fungal pathogen [112].

Analysis of the enzymes from the IMP branchpoint to either adenine or guanine synthesis has been carried out for four enzymes in *C. neoformans*: adenylosuccinate synthetase (ADSS) (CNAG_02858, *ADE12*), adenylosuccinate lyase (ADSL) (CNAG_03270, *ADE13*), inosine monophosphate dehydrogenase (IMPDH) (CNAG_00441, *IMD1*) and guanine monophosphate synthase (GMP synthase) (CNAG_01877, *GUA1*) [108,109,113,114]. Deletion of the genes encoding these enzymes leads to strains that are purine auxotrophs, are attenuated for virulence factor production and are avirulent in a murine inhalation model, contrasting starkly with the salvage mutants and highlighting the importance of de novo purine biosynthesis during infection [108,109,113,114]. Biochemical and structural analyses have determined potential differences between these fungal enzymes and their human counterparts that may be exploited in the development of fungal-specific therapeutics [108,109,111,112,113,114].

## 9. Purine Biosynthesis as an Antifungal Drug Target

Since the 1940s, the synthesis of purines has been an important biochemical pathway in the discovery of novel drugs [80,115,116]. The enzymatic activities of the purine biosynthesis pathway have been particularly useful targets in the development or discovery of antibiotic, anticancer and immunosuppressive agents, such as hadacidin, mercaptopurine, and mycophenolic acid (MPA) [117,118,119,120,121,122]. 

MPA, an inhibitor of the rate-limiting enzyme IMPDH in de novo synthesis of guanosine nucleotides, has been shown to have activity against *C. albicans*, *A. fumigatus* and *C. neoformans* [70,108,123,124]. Mode of action studies have determined that MPA binds to the site of the mobile flap of IMPDH and prevents formation of the closed enzyme conformation [125]. Studies of *C. neoformans* IMPDH have shown that, while it is inhibited by MPA, unlike mammalian IMPDH, this drug is able to bind to all conformations of the fungal IMPDH and not exclusively to the open conformation [108]. Interestingly, only limited inhibition of *A. fumigatus* IMPDH by MPA occurs, which is perhaps unsurprising given that MPA is produced by several *Penicillium* species commonly found in the same environments, and *A. fumigatus*, therefore, may have developed some resistance to the compound produced by competing species in its environmental niche [126]. Unfortunately, MPA itself cannot be used as an antifungal agent in the clinic against opportunistic pathogens due to its immunosuppressive activity; however, investigating this drug for activity against fungal enzymes is proof of principle that the enzyme of these pathways can be targeted by inhibitors of purine biosynthesis and may be a starting point for fungal specific inhibitor development.

The l-aspartate analog hadacidin has been identified as an antibiotic and anticancer agent that targets ADS synthetase [117,127]. First isolated from *Penicillium frequentans*, this compound exhibits 100% inhibition against *Eschrichia coli* as well as excellent clinical activity against human adenocarcinoma [128]. However, in fungi, this compound does not show antifungal activity against *C. neoformans* or *A. fumigatus*, and whilst there is some inhibition of *C. albicans* growth, this is limited [109]. In enzyme kinetics assays, hadacidin cannot fully inhibit *C. neoformans* ADS synthetase, but, again, this compound could serve as a basis for the development of antimycotics that act via ADS synthetase [109]. While little, if anything, is known about the antifungal activity of other purine biosynthesis inhibitors such as mercaptopurine (which closely resembles hypoxanthine and adenine and targets the HGPRT enzyme), the known activities of MPA and hadacidin along with the available crystal structures of their targets suggests that purine biosynthesis has the potential to be a valuable target for future antimycotic development. 

## 10. Conclusions

While the salvage of environmental purines, the synthesis of de novo purine nucleotides and the breakdown of purines to their simplest form, ammonia, are common to the fungi that pose the most consistent major threat to humans, these processes are not all essential during the infection process. Scavenging purines from their environmental niche likely confers a selective advantage to *A. fumigatus*, *C. albicans* and *C. neoformans*. However, during infection, the de novo biosynthesis pathway is essential, likely due to pressures such as rapid proliferation, host immune defenses, and differences in purine availability. Deletion or disruption of enzymes from the de novo purine pathway in all three species are associated with either avirulence or reduced virulence of strains, making these an attractive antifungal drug target. 

## Figures and Tables

**Table 1:** Concentration of purines (μM unless indicated) from the habitats of *C. albicans*, *A. fumigatus* and *C. neoformans*.

Source of purine	Adenine	Guanine	Xanthine	Hypoxanthine	Inosine	Reference
Average meal ^1^ (per gram)	0.9	1.0	1.8	0.02	ND	[58]
Human Blood serum	0.4	97	20	172	168	[60]
Human Cerebral spinal fluid	0.2	0.5	2.4	3.9	0.6	[29,30]
Human Intracellular	1.5	97	ND	370	211	[60]
Plant matter average ^2^	0.4 μg/mL	1.3 μg/mL	0.8 μg/mL	1.0 μg/mL	1.2 μg/mL	[61,62]
Soil average ^3^	19 M %	19 M %	ND	ND	ND	[63]

ND for no data. ^1^ Average meal defined as 100g rice, 75g carrot, 75g peas, 100g chicken [58]. ^2^ Average plant matter concentration from of *A. vaginalis*, *Z. jujuba*, *Z. jujuba* var. *spinosa* and *Z. mauritiana* plants [61,62]. ^3^ An average soil concentration was determined as proportion of purine relative to the g of air-dried soil from different locations [63].

## References

[1] Clayton M. (2006). Three newly published albums of seventeenth-century mycological drawings. Mycologist.

[2] Micheli P.A. Nova Plantarum Genera. https://books.google.co.uk/books?hl=en&lr=&id=hIozjzXbJQEC&oi=fnd&pg=PA1&dq=Nova+Plantarum+Genera&ots=5W-BBlkw6g&sig=3SM31sAcacKOc5MUJzkwt1zbfcY#v=onepage&q=Nova%20Plantarum%20Genera&f=false.

[3] Linné C.V. (1758). Systema Naturæ Per Regna Tria Naturæ, Secundum Classes, Ordines, Genera, Species, Cum Characteribus, Differentiis, Synonymis, Locis.

[4] Persoon C.H., Lünemann G.H. (1801). Synopsis Methodica Fungorum.

[5] Fries E.M. (1821). Systema Mycologicum, Sistens Fungorum Ordines, Genera et Species, huc Usque Cognitas.

[6] Petersen R.H., Knudsen H. (2015). The mycological legacy of Elias Magnus Fries. IMA Fungus.

[7] Whittaker R.H. (1969). New concepts of kingdoms or organisms. Evolutionary relations are better represented by new classifications than by the traditional two kingdoms. Science.

[8] Hawksworth D.L. (1984). Recent changes in the international rules affecting the nomenclature of fungi. Microbiol. Sci..

[9] Hawksworth D.L. (1991). The fungal dimension of biodiversity—Magnitude, significance, and conservation. Mycol. Res..

[10] Blackwell M. (2011). The fungi: 1, 2, 3... 5.1 million species?. Am. J. Bot..

[11] Burgaud G., Le Calvez T., Arzur D., Vandenkoornhuyse P., Barbier G. (2009). Diversity of culturable marine filamentous fungi from deep-sea hydrothermal vents. Environ. Microbiol..

[12] Goncalves V.N., Cantrell C.L., Wedge D.E., Ferreira M.C., Soares M.A., Jacob M.R., Oliveira F.S., Galante D., Rodrigues F., Alves T.M. (2016). Fungi associated with rocks of the atacama desert: Taxonomy, distribution, diversity, ecology and bioprospection for bioactive compounds. Environ. Microbiol..

[13] Lyakh S.P., Kozlova T.M., Salivonik S.M. (1983). Effect of periodic freezing and thawing on cells of the Antarctic black yeast nadsoniella-nigra var hesuelica. Microbiology.

[14] Badiee P., Hashemizadeh Z. (2014). Opportunistic invasive fungal infections: Diagnosis & clinical management. Indian J. Med. Res..

[15] Drgona L., Khachatryan A., Stephens J., Charbonneau C., Kantecki M., Haider S., Barnes R. (2014). Clinical and economic burden of invasive fungal diseases in Europe: Focus on pre-emptive and empirical treatment of *Aspergillus* and *Candida* species. Eur. J. Clin. Microbiol. Infect. Dis..

[16] Zilberberg M.D., Shorr A.F., Kollef M.H. (2008). Secular trends in candidemia-related hospitalization in the United States, 2000–2005. Infect. Control Hosp. Epidemiol..

[17] Wenzel R.P., Gennings C. (2005). Bloodstream infections due to *Candida* species in the intensive care unit: Identifying especially high-risk patients to determine prevention strategies. Clin. Infect. Dis..

[18] Kosmidis C., Denning D.W. (2015). The clinical spectrum of pulmonary aspergillosis. Thorax.

[19] Perfect J.R., Dismukes W.E., Dromer F., Goldman D.L., Graybill J.R., Hamill R.J., Harrison T.S., Larsen R.A., Lortholary O., Nguyen M.H. (2010). Clinical practice guidelines for the management of cryptococcal disease: 2010 update by the infectious diseases society of America. Clin. Infect. Dis..

[20] Rénon L. (1897). Étude Sur L'aspergillose Chez Les Animaux Et Chez L’homme.

[21] Staib F. (1962). Vogelkot, ein nahrsubstrat fur die gattung cryptococcus. Zentralbl. Bakteriol..

[22] Staib F., Seeliger H.P. (1966). A new selective medium for the isolation of *C. neoformans* from fecal material and from soil. Ann. Inst. Pasteur.

[23] Emmons C.W. (1955). Saprophytic sources of *Cryptococcus neoformans* associated with the pigeon (*Columba livia*). Am. J. Hyg..

[24] Badiee P., Kordbacheh P., Alborzi A., Zeini F., Mirhendy H., Mahmoody M. (2005). Fungal infections in solid organ recipients. Exp. Clin. Transpl..

[25] Nucci M., Anaissie E. (2001). Revisiting the source of candidemia: Skin or gut?. Clin. Infect. Dis..

[26] Braun E.J. (1999). Integration of organ systems in avian osmoregulation. J. Exp. Zool..

[27] Yamaoka N., Kaneko K., Kudo Y., Aoki M., Yasuda M., Mawatari K., Nakagomi K., Yamada Y., Yamamoto T. (2010). Analysis of purine in purine-rich cauliflower. Nucleosides Nucleotides Nucleic Acids.

[28] Schreiner O., Shorey E.C. (1910). Pyrimidine derivatives and purine bases in soils. J. Biol. Chem..

[29] Rodriguez-Nunez A., Camina F., Lojo S., Rodriguez-Segade S., Castro-Gago M. (1993). Concentrations of nucleotides, nucleosides, purine bases and urate in cerebrospinal fluid of children with meningitis. Acta Paediatr..

[30] Fairbanks L.D., Harris J.C., Duley J.A., Simmonds H.A. (2004). Nucleotide degradation products in cerebrospinal fluid (CSF) in inherited and acquired pathologies. Nucleosides Nucleotides Nucleic Acids.

[31] Eells J.T., Spector R. (1983). Purine and pyrimidine base and nucleoside concentrations in human cerebrospinal fluid and plasma. Neurochem. Res..

[32] Fischer E., Ach L. (1985). Neue synthese der harnsäure und ihrer methylderivate. Untersuchungen in der Puringruppe.

[33] Rosemeyer H. (2004). The chemodiversity of purine as a constituent of natural products. Chem. Biodivers..

[34] Oparin A.I., Morgulis S. (1938). The Origin of Life.

[35] Miller S.L., Urey H.C. (1959). Organic compound synthesis on the primitive earth. Science.

[36] Marzluf G.A. (1997). Genetic regulation of nitrogen metabolism in the fungi. Microbiol. Mol. Biol. Rev..

[37] Lee I.R., Chow E.W., Morrow C.A., Djordjevic J.T., Fraser J.A. (2011). Nitrogen metabolite repression of metabolism and virulence in the human fungal pathogen *Cryptococcus neoformans*. Genetics.

[38] Griffin D.H. (1994). Fungal Physiology.

[39] Vogels G.D., Van der Drift C. (1976). Degradation of purines and pyrimidines by microorganisms. Bacteriol. Rev..

[40] Sumrada R., Cooper T.G. (1977). Allantoin transport in *Saccharomyces cerevisiae*. J. Bacteriol..

[41] Spanu P.D. (2012). The genomics of obligate (and nonobligate) biotrophs. Annu. Rev. Phytopathol..

[42] Cisse O.H., Pagni M., Hauser P.M. (2014). Comparative genomics suggests that the human pathogenic fungus *Pneumocystis jirovecii* acquired obligate biotrophy through gene loss. Genome Biol. Evol..

[43] Ljungdahl P.O., Daignan-Fornier B. (2012). Regulation of amino acid, nucleotide, and phosphate metabolism in saccharomyces cerevisiae. Genetics.

[44] Pantazopoulou A., Diallinas G. (2007). Fungal nucleobase transporters. FEMS Microbiol. Rev..

[45] Stouthamer A.H. (1973). A theoretical study on the amount of ATP required for synthesis of microbial cell material. Antonie van Leeuwenhoek.

[46] Phillips R., Milo R. (2009). A feeling for the numbers in biology. Proc. Natl. Acad. Sci. USA.

[47] Dean P., Hirt R.P., Embley T.M. (2016). Microsporidia: Why make nucleotides if you can steal them?. PLoS Pathog..

[48] Hirt R.P., Logsdon J.M., Healy B., Dorey M.W., Doolittle W.F., Embley T.M. (1999). Microsporidia are related to fungi: Evidence from the largest subunit of RNA polymerase II and other proteins. Proc. Natl. Acad. Sci. USA.

[49] Galenus (2014). Commentary on Hippocrates’ Epidemics Book I, Parts I-III.

[50] Galen, Vagelpohl U., Swain S. (2016). Commentary on Hippocrates’ Epidemics Book II. Parts I-VI.

[51] Knoke M., Bernhardt H. (2006). The first description of an oesophageal candidosis by Bernhard von Langenbeck in 1839. Mycoses.

[52] Barnett J.A. (2004). A history of research on yeasts 8: Taxonomy. Yeast.

[53] Magill S.S., Edwards J.R., Bamberg W., Beldavs Z.G., Dumyati G., Kainer M.A., Lynfield R., Maloney M., McAllister-Hollod L., Nadle J. (2014). Multistate point-prevalence survey of health care-associated infections. N. Engl. J. Med..

[54] Buffo J., Herman M.A., Soll D.R. (1984). A characterization of pH-regulated dimorphism in *Candida albicans*. Mycopathologia.

[55] Lu Y., Su C., Wang A., Liu H. (2011). Hyphal development in *Candida albicans* requires two temporally linked changes in promoter chromatin for initiation and maintenance. PLoS Biol..

[56] Klengel T., Liang W.J., Chaloupka J., Ruoff C., Schroppel K., Naglik J.R., Eckert S.E., Mogensen E.G., Haynes K., Tuite M.F. (2005). Fungal adenylyl cyclase integrates CO_2_ sensing with cAMP signaling and virulence. Curr. Biol..

[57] Lu Y., Su C., Liu H. (2014). *Candida albicans* hyphal initiation and elongation. Trends Microbiol..

[58] Kaneko K., Aoyagi Y., Fukuuchi T., Inazawa K., Yamaoka N. (2014). Total purine and purine base content of common foodstuffs for facilitating nutritional therapy for gout and hyperuricemia. Biol. Pharm. Bull..

[59] Karasawa Y., Ishi I.T., Kubota T. (1991). Absorption and metabolism of purines by the small intestine of the chicken. Comp. Biochem. Physiol. A Comp. Physiol..

[60] Traut T.W. (1994). Physiological concentrations of purines and pyrimidines. Mol. Cell. Biochem..

[61] Liu H., Lei M., Liang X., Jiang Z., Guo X. (2014). Simultaneous determination of three purines in *Alysicarpus vaginalis* (l.) dc. By hollow fiber-based liquid-phase microextraction combined with high-performance liquid chromatography. Biomed. Chromatogr..

[62] Guo S., Duan J.A., Qian D., Wang H., Tang Y., Qian Y., Wu D., Su S., Shang E. (2013). Hydrophilic interaction ultra-high performance liquid chromatography coupled with triple quadrupole mass spectrometry for determination of nucleotides, nucleosides and nucleobases in *Ziziphus* plants. J. Chromatogr. A.

[63] Cortez J., Schnitzer M. (1979). Purines and pyrimidines in soils and humic substances. Soil Sci. Soc. Am. J..

[64] Tebung W.A., Choudhury B.I., Tebbji F., Morschhauser J., Whiteway M. (2016). Rewiring of the ppr1 zinc cluster transcription factor from purine catabolism to pyrimidine biogenesis in the *Saccharomycetaceae*. Curr. Biol..

[65] Vylkova S., Lorenz M.C. (2017). Phagosomal neutralization by the fungal pathogen *Candida albicans* induces macrophage pyroptosis. Infect. Immun..

[66] Balish E., Svihla G. (1968). Ultraviolet microscopy of purines and amino acids in the vacuole of *Candida albicans*. J. Bacteriol..

[67] Rodrigues L., Russo-Abrahao T., Cunha R.A., Goncalves T., Meyer-Fernandes J.R. (2016). Characterization of extracellular nucleotide metabolism in *Candida albicans*. FEMS Microbiol. Lett..

[68] Poulter R.T., Rikkerink E.H. (1983). Genetic analysis of red, adenine-requiring mutants of *Candida albicans*. J. Bacteriol..

[69] Donovan M., Schumuke J.J., Fonzi W.A., Bonar S.L., Gheesling-Mullis K., Jacob G.S., Davisson V.J., Dotson S.B. (2001). Virulence of a phosphoribosylaminoimidazole carboxylase-deficient *Candida albicans* strain in an immunosuppressed murine model of systemic candidiasis. Infect. Immun..

[70] Rodriguez-Suarez R., Xu D., Veillette K., Davison J., Sillaots S., Kauffman S., Hu W., Bowman J., Martel N., Trosok S. (2007). Mechanism-of-action determination of GMP synthase inhibitors and target validation in *Candida albicans* and *Aspergillus fumigatus*. Chem. Biol..

[71] Jiang L., Zhao J., Guo R., Li J., Yu L., Xu D. (2010). Functional characterization and virulence study of ADE8 and GUA1 genes involved in the de novo purine biosynthesis in *Candida albicans*. FEMS Yeast Res..

[72] Banerjee D., Burkard L., Panepinto J.C. (2014). Inhibition of nucleotide biosynthesis potentiates the antifungal activity of amphotericin b. PLoS ONE.

[73] Plaignaud M. (1791). Observation sur un fungus du sinus maxillaire. J. Méd. Chir. Pharm..

[74] Schmidt A., Schmidt D.I. (1999). J.B. Georg W. Fresenius and the description of the species *Aspergillus fumigatus* in 1863. Contrib. Microbiol..

[75] Ruchlemer R., Yinnon A.M., Hershko C. (1996). Changes in the natural history of invasive pulmonary aspergillosis in neutropenic leukemic patients. Isr. J. Med. Sci..

[76] Cohen J., Denning D.W., Viviani M.A. (1993). Epidemiology of invasive aspergillosis in European cancer centres. Eortc invasive fungal infections cooperative group. Eur. J. Clin. Microbiol. Infect. Dis..

[77] Groll A.H., Shah P.M., Mentzel C., Schneider M., Just-Nuebling G., Huebner K. (1996). Trends in the postmortem epidemiology of invasive fungal infections at a university hospital. J. Infect..

[78] Latge J.P. (1999). Aspergillus fumigatus and aspergillosis. Clin. Microbiol. Rev..

[79] Bodey G., Bueltmann B., Duguid W., Gibbs D., Hanak H., Hotchi M., Mall G., Martino P., Meunier F., Milliken S. (1992). Fungal infections in cancer patients: An international autopsy survey. Eur. J. Clin. Microbiol. Infect. Dis..

[80] Elion G.B. (1985). An overview of the role of nucleosides in chemotherapy. Adv. Enzyme Regul..

[81] Patel R., Paya C.V. (1997). Infections in solid-organ transplant recipients. Clin. Microbiol. Rev..

[82] Denning D.W. (1995). Issues in the management of invasive aspergillosis. Ann. Med. Interne.

[83] Denning D.W. (1996). Therapeutic outcome in invasive aspergillosis. Clin. Infect. Dis..

[84] Dagenais T.R., Keller N.P. (2009). Pathogenesis of *Aspergillus fumigatus* in invasive aspergillosis. Clin. Microbiol. Rev..

[85] Kwon-Chung K.J., Sugui J.A. (2013). *Aspergillus fumigatus*—What makes the species a ubiquitous human fungal pathogen?. PLoS Pathog..

[86] Matsuyama H., Amaya F., Hashimoto S., Ueno H., Beppu S., Mizuta M., Shime N., Ishizaka A., Hashimoto S. (2008). Acute lung inflammation and ventilator-induced lung injury caused by ATP via the p2y receptors: An experimental study. Respir. Res..

[87] Gournas C., Oestreicher N., Amillis S., Diallinas G., Scazzocchio C. (2011). Completing the purine utilisation pathway of *Aspergillus nidulans*. Fungal Genet. Biol..

[88] Scazzocchio C. (1994). The purine degradation pathway, genetics, biochemistry and regulation. Prog. Ind. Microbiol..

[89] Scazzocchio C., Darlington A.J. (1967). The genetic control of xanthine dehydrogenase and urate oxidase synthess in *Aspergillus nidulans*. Bull. Soc. Chim. Biol..

[90] Scazzocchio C., Sdrin N., Ong G. (1982). Positive regulation in a eukaryote, a study of the uaY gene of *Aspergillus nidulans*: I. Characterization of alleles, dominance and complementation studies, and a fine structure map of the uaY--oxpa cluster. Genetics.

[91] Oestreicher N., Ribard C., Scazzocchio C. (2008). The nada gene of *Aspergillus nidulans*, encoding adenine deaminase, is subject to a unique regulatory pattern. Fungal Genet. Biol..

[92] Thykaer J., Andersen M.R., Baker S.E. (2009). Essential pathway identification: From *in silico* analysis to potential antifungal targets in *Aspergillus fumigatus*. Med. Mycol..

[93] Sanfelice F. (1895). Sull’azione patogena dei bastomiceti [on the action of pathogenic bastomiceti]. Ann. Inst Igien. Univ. Roma.

[94] Knoke M., Schwesinger G. (1994). One hundred years ago: The history of cryptococcosis in greifswald. Medical mycology in the nineteenth century. Mycoses.

[95] Dix N.J., Webster J. (1995). Fungal Ecology.

[96] Baldrian P., Valaskova V. (2008). Degradation of cellulose by basidiomycetous fungi. FEMS Microbiol. Rev..

[97] Stubblefield S.P., Taylor T.N., Beck C.B. (1985). Studies of paleozoic fungi. 4. Wood-decaying fungi in callixylon-newberryi from the upper devonian. Am. J. Bot..

[98] Rao K.P., Gopalakrishnareddy T. (1962). Nitrogen excretion in arachnids. Comp. Biochem. Physiol..

[99] Schmidt G., Liss M., Thannhauser S.J. (1955). Guanine, the principal nitrogenous component of the excrements of certain spiders. Biochim. Biophys. Acta.

[100] Quilter J. (2002). Moche politics, religion, and warfare. J. World Prehist..

[101] International Union of American Republics (1909). Bulletin of the International Union of the American Republics.

[102] Magnus (1844). Ueber das vorkommen von xanthicoxyd im guano. Ann. Chem. Pharm..

[103] Nielsen K., De Obaldia A.L., Heitman J. (2007). *Cryptococcus neoformans* mates on pigeon guano: Implications for the realized ecological niche and globalization. Eukaryot. Cell.

[104] Lee I.R., Yang L., Sebetso G., Allen R., Doan T.H., Blundell R., Lui E.Y., Morrow C.A., Fraser J.A. (2013). Characterization of the complete uric acid degradation pathway in the fungal pathogen *Cryptococcus neoformans*. PLoS ONE.

[105] Shi M., Li S.S., Zheng C., Jones G.J., Kim K.S., Zhou H., Kubes P., Mody C.H. (2010). Real-time imaging of trapping and urease-dependent transmigration of *Cryptococcus neoformans* in mouse brain. J. Clin. Investig..

[106] Olszewski M.A., Noverr M.C., Chen G.H., Toews G.B., Cox G.M., Perfect J.R., Huffnagle G.B. (2004). Urease expression by *Cryptococcus neoformans* promotes microvascular sequestration, thereby enhancing central nervous system invasion. Am. J. Pathol..

[107] Cox G.M., Mukherjee J., Cole G.T., Casadevall A., Perfect J.R. (2000). Urease as a virulence factor in experimental cryptococcosis. Infect. Immun..

[108] Morrow C.A., Valkov E., Stamp A., Chow E.W., Lee I.R., Wronski A., Williams S.J., Hill J.M., Djordjevic J.T., Kappler U. (2012). *De novo* GTP biosynthesis is critical for virulence of the fungal pathogen *Cryptococcus neoformans*. PLoS Pathog..

[109] Blundell R.D., Williams S.J., Arras S.D.M., Chitty J.L., Blake K.L., Ericsson D.J., Tibrewal N., Rohr J., Koh Y.Q.A.E., Kappler U. (2016). Disruption of de novo adenosine triphosphate (ATP) biosynthesis abolishes virulence in *Cryptococcus neoformans*. ACS Infect. Dis..

[110] Arras S.D., Chitty J.L., Blake K.L., Schulz B.L., Fraser J.A. (2015). A genomic safe haven for mutant complementation in *Cryptococcus neoformans*. PLoS ONE.

[111] Perfect J.R., Toffaletti D.L., Rude T.H. (1993). The gene encoding phosphoribosylaminoimidazole carboxylase (ADE2) is essential for growth of *Cryptococcus neoformans* in cerebrospinal fluid. Infect. Immun..

[112] Firestine S.M., Misialek S., Toffaletti D.L., Klem T.J., Perfect J.R., Davisson V.J. (1998). Biochemical role of the *Cryptococcus neoformans* ADE2 protein in fungal de novo purine biosynthesis. Arch. Biochem. Biophys..

[113] Chitty J.L., Tatzenko T.L., Williams S.J., Koh Y.Q., Corfield E.C., Butler M.S., Robertson A.A., Cooper M.A., Kappler U., Kobe B. (2017). GMP synthase is required for virulence factor production and infection by *Cryptococcus neoformans*. J. Biol. Chem..

[114] Chitty J.L., Blake K.L., Blundell R.D., Koh Y.Q.A.E., Thompson M., Robertson A.A.B., Butler M.S., Cooper M.A., Kappler U., Williams S.J. (2017). *Cryptococcus neoformans* ADS lyase in an enzyme essential for virulence whose crystal structure reveals features exploitable in antifungal drug design. J. Biol. Chem..

[115] Hitchings G.H., Elion G.B., Vanderwerff H. (1948). 2-aminopurine as a purine antagonist. Fed. Proc..

[116] Elion G.B. (1989). Nobel lecture. The purine path to chemotherapy. Biosci. Rep..

[117] Shigeura H.T., Gordon C.N. (1962). Hadacidin, a new inhibitor of purine biosynthesis. J. Biol. Chem..

[118] Christopherson R.I., Lyons S.D., Wilson P.K. (2002). Inhibitors of de novo nucleotide biosynthesis as drugs. Acc. Chem. Res..

[119] Skipper H.E., Thomson J.R., Elion G.B., Hitchings G.H. (1954). Observations on the anticancer activity of 6-mercaptopurine. Cancer Res..

[120] Mendelsohn L.G., Shih C., Schultz R.M., Worzalla J.F. (1996). Biochemistry and pharmacology of glycinamide ribonucleotide formyltransferase inhibitors: Ly309887 and lometrexol. Investig. New Drugs.

[121] Franklin T.J., Cook J.M. (1969). The inhibition of nucleic acid synthesis by mycophenolic acid. Biochem. J..

[122] Sweeney M.J., Hoffman D.H., Esterman M.A. (1972). Metabolism and biochemistry of mycophenolic acid. Cancer Res..

[123] Kohler G.A., Gong X., Bentink S., Theiss S., Pagani G.M., Agabian N., Hedstrom L. (2005). The functional basis of mycophenolic acid resistance in *Candida albicans* IMP dehydrogenase. J. Biol. Chem..

[124] Mezger M., Wozniok I., Blockhaus C., Kurzai O., Hebart H., Einsele H., Loeffler J. (2008). Impact of mycophenolic acid on the functionality of human polymorphonuclear neutrophils and dendritic cells during interaction with *Aspergillus fumigatus*. Antimicrob. Agents Chemother..

[125] Guillen Schlippe Y.V., Riera T.V., Seyedsayamdost M.R., Hedstrom L. (2004). Substitution of the conserved arg-tyr dyad selectively disrupts the hydrolysis phase of the IMP dehydrogenase reaction. Biochemistry.

[126] Schneweis I., Meyer K., Hormansdorfer S., Bauer J. (2000). Mycophenolic acid in silage. Appl. Environ. Microbiol..

[127] Demain A.L. (1966). Mode of action of hadacidin in the growing bacterial cell. Nature.

[128] Tibrewal N., Elliott G.I. (2011). Evaluation of hadacidin analogues. Bioorg. Med. Chem. Lett..

